# In vitro assessment of effect of initial specimen diversion device on detection of central venous catheter contamination or colonization

**DOI:** 10.1017/ice.2024.220

**Published:** 2025-02

**Authors:** Mark E. Rupp, Paul D. Fey, Elizabeth Lyden, Luke Handke

**Affiliations:** 1 Division of Infectious Diseases, University of Nebraska Medical Center, Omaha, NE, USA; 2 Department of Pathology, Microbiology, and Immunology, University of Nebraska Medical Center, Omaha, NE, USA; 3 Department of Biostatistics, University of Nebraska Medical Center, Omaha, NE, USA

## Abstract

The role of initial specimen diversion devices (ISDDs) in preventing contamination of central venous catheter (CVC) blood cultures is undefined. A model to simulate CVC colonization and contamination compared standard cultures with ISDD technique. ISDD detected 100% of colonized CVCs while decreasing false-positive cultures from 36% to 16%.

## Introduction

Blood cultures are commonly used and critically important.^1^ 1%–5% of blood cultures are contaminated by skin-residing commensal organisms, resulting in adverse outcomes.^1–3^

Catheter-related bloodstream infections (CRBSIs) are common and result in morbidity and excess cost.^1–4^ Diagnosis of CRBSI often requires cultures from the vascular catheter and peripheral blood.^5^ Cultures obtained from vascular catheters are more likely than peripheral cultures to be contaminated,^6^ often leading to unnecessary catheter removal.

Initial specimen diversion devices (ISDDs) cost-efficiently decrease blood culture contamination.^7^ It is unknown whether ISDD technology is helpful in decreasing contamination of central venous catheter (CVC)-drawn cultures. We developed in vitro models of CVC contamination and colonization to characterize the utility of the ISDD in detecting CVC colonization while minimizing culture contamination.

## Methods: (Also see Supplemental Materials)

### CVC colonization

CVC (Arrow triple-lumen, Teleflex) colonization was simulated by filling the lumen with Trypticase Soy Broth (TSB) containing 10 colony-forming units (CFUs) of *S. epidermidis* 1457/pCM29 (*ica*-positive, biofilm-producing, expressing green fluorescent protein (GFP)). The CVCs, maintained in sterile catheter shields (Cath–Gard, Teleflex), were incubated overnight at 37°C. 50 CVCs were sampled by standard or ISDD method.

### Catheter contamination

Catheter connector (MaxZero™, Becton Dickinson) contamination was simulated by inoculation of 10 CFUs of *S. epidermidis* in 50 µL of TSB onto the diaphragm of the CVC connector and allowing it to air dry. Fifty CVCs were sampled by standard or ISDD method.

### Blood culture

Blood cultures were simulated by drawing sterile phosphate-buffered saline (PBS) through the CVC. In the standard method, the ISDD (SteriPath Blood Collection System, Magnolia Medical Technologies) was pre-engaged (diversion chamber closed) and then connected to the CVC. For the ISDD method, the ISDD was engaged to divert the initial 1.5–2 mL of PBS. For both methods, a Vacutainer (Becton Dickinson) was used to collect 1 mL of PBS for quantitative culture. Two 10 mL samples were then collected into BACTEC Plus Aerobic/F blood bottles (Becton Dickinson). The bacterial titer in CFU/mL was determined. Bottles were incubated in a BACTEC FX instrument (Becton Dickinson) and monitored for 5 days. Time-to-positivity (TTP) was recorded.

### Dose-ranging preliminary study

To establish inoculum levels to simulate CVC connector contamination and catheter colonization, a dose-ranging study was conducted using 3 CVCs per inoculum group: 10^1^ CFU–10^3^ CFU in colonization model; 10^1^ CFU–10^4^ CFU in contamination model.

### Scanning electron microscopy (SEM)

(See supplemental materials).

### Statistical analysis

Fisher’s exact test compared the proportion of positive cultures between the ISDD and standard methods. Wilcoxon rank sum test compared the median CFU/mL between the blood culture methods.

## Results

### Dose-ranging study

Colonization: All inoculum levels (10^1^–10^3^ CFU) resulted in 100% blood culture positivity. Table 1 details quantitative culture and TTP data. For the full study, the lowest inoculum (10^1^ CFU) that reliably resulted in colonized CVCs was used.

**Table 1.** Dose-ranging study. Quantitative culture results and blood culture time-to-positivity for colonization model and contamination model

	Blood culture method	
Inoculum 10^1^ CFU	ISDD	Standard
**Colonization model**		
Quantitative blood culture (mean CFU/mL + SD)	1.4 × 10^5^ ± 3.4 × 10^4^	1.8 × 10^7^ ± 6.1 × 10^6^
1^st^ bottle TTP (hours) (mean + SD)	10.97 ± 0.93	7.75 ± 0.33
2^nd^ bottle TTP (hours) (mean + SD)	12.17 ± 0.39	10.2 ± 0.45
**Inoculum 10** ^**2**^ **CFU**		
Quantitative blood culture (mean CFU/mL + SD)	8.6 × 10^6^ ± 3.7 × 10^6^	9.5 ×10^7^ ± 3.7 × 10^7^
1^st^ bottle TTP (hours) (mean + SD)	7.7 ± 1.0	6.1 ± 0.2
2^nd^ bottle TTP (hours) (mean + SD)	10.0 ± 1.6	8.24 ± 0.7
**Inoculum 10** ^**3**^ **CFU**		
Quantitative blood culture (mean CFU/mL + SD)	4.6 × 10^7^ ± 1.2 ×10^7^	3.0 × 10^8^ ± 3.0 × 10^7^
1^st^ bottle TTP (hours) (mean + SD)	5.06 ± 0.0	5.0 ± 0.1
2^nd^ bottle TTP (hours) (mean + SD)	6.17 ± 0.6	6.0 ± 0.8
**Contamination model**		
Quantitative blood culture (mean CFU/mL + SD)	0 ± 0	10 ± 10
1^st^ bottle TTP (hours) (mean + SD)	22.4 (1 positive)	22.1 ± 0.7 (2 positive)
2^nd^ bottle TTP (hours) (mean + SD)	0 (0 positive)	0 (0 positive)
**Inoculum 10** ^**2**^ **CFU**		
Quantitative blood culture (mean CFU/mL + SD)	10 ± 10	16.7 ± 5.8
1^st^ bottle TTP (hours) (mean + SD)	21.1 ± 0.5 (3 positive)	19.9 ± 0.3 (3 positive)
2^nd^ bottle TTP (hours) (mean + SD)	0 (0 positive)	0 (0 positive)
**Inoculum 10** ^**3**^ **CFU**		
Quantitative blood culture (mean CFU/mL + SD)	50 ± 86	180 ± 112
1^st^ bottle TTP (hours) (mean + SD)	18.7 ± 0.3 (3 positive)	18.7 ± 0.6 (3 positive)
2^nd^ bottle TTP (hours) (mean + SD)	21.5 ± 0.7 (3 positive)	20.9 ± 0.5 (3 positive)
**Inoculum 10** ^**4**^ **CFU**		
Quantitative blood culture (mean CFU/mL + SD)	343 ± 421	7.1 × 10^3^ ± 9.6 × 10^3^
1^st^ bottle TTP (hours) (mean + SD)	17.9 ± 0.7 (3 positive)	16.4 ± 0.4 (3 positive)
2^nd^ bottle TTP (hours) (mean + SD)	19.6 ± 0.1 (3 positive)	18.7 ± 0.1 (3 positive)

Note. CFU, colony-forming units; ISDD, initial specimen diversion device; TTP, time-to-positivity.

Contamination: At the 10^1^ level, a differentiation was evident between ISDD and standard techniques. At larger inoculum (10^2^–10^4^ CFU), both the ISDD and standard cultures were positive. For the full study, the 10^1^ CFU level was chosen.

### Full study

Catheter colonization: All simulated blood cultures yielded bacterial growth in the standard and ISDD arms. There was a lower number of bacteria in the ISDD cultures versus standard cultures (1.17 × 10^5^ CFU/mL ± 1.85 × 10^5^ (SD) vs 3.96 × 10^6^ CFU/mL ± 4.97 × 10^6^, respectively) (*P* < 0.0001). There was a significant difference in TTP for the initial bottle (12.51 h ± 1.61 h vs 10.02 h ± 1.03 h) (*P* < 0.0001) and the second bottle (14.14 h ± 1.81 h vs. 12.92 h ± 1.18 h) (*P* = 0.017) for ISDD and standard cultures, respectively.

Connector contamination: 4 of 25 (16%) ISDD cultures and 9 of 25 (36%) standard cultures yielded bacteria (*P* = 0.196). 0 of 25 quantitative ISDD cultures recovered bacteria, and 2 of 25 standard cultures yielded growth. There was not a significant difference in TTP between ISDD and standard cultures. First bottle positivity was 23.76 h ±1.83 h versus 22.94 h ±1.2 h (*P* = 0.247) for the ISDD and standard cultures, respectively. Second bottle positivity was 28.4 h (only 1 positive bottle) versus 22.56 h ±1.26 h (*P* = 0.54) for the ISDD and standard cultures, respectively.

Inadvertent contamination was excluded with all arising colonies demonstrating GFP production.

**Figure 1.**
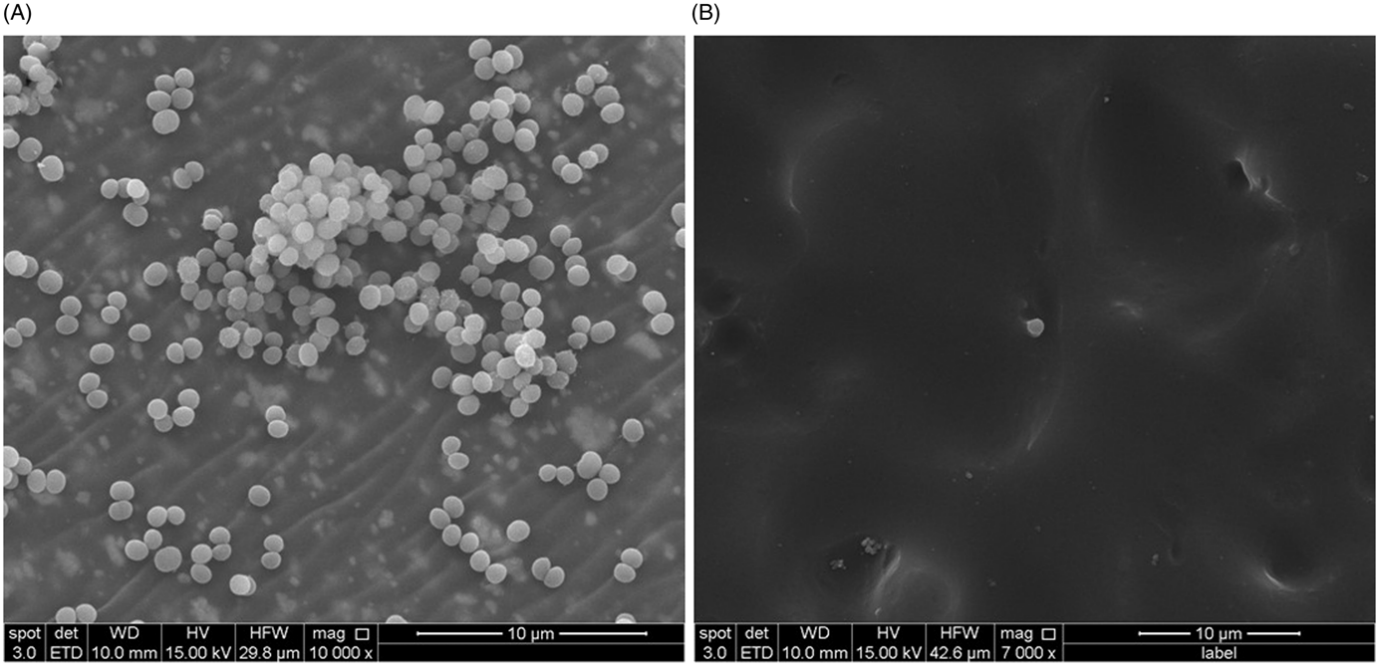
(A) Representative scanning electron micrograph of colonized catheter demonstrating numerous intralumenal adherent staphylococci. (B) Representative scanning electron micrograph of contaminated diaphragmatic surface of CVC connector valve demonstrating rare adherent staphylococci.

Four colonized CVCs and two contaminated connectors were examined by scanning electron microscopy (SEM). All colonized catheters exhibited widespread adherent staphylococci (Figure 1A), while only rare bacteria were visualized on the contaminated connectors (Figure 1B).

## Discussion

ISDD prevents contamination of peripheral blood cultures.^7^ CVC blood cultures are more likely to be contaminated than peripheral cultures.^6^

Whether ISDD limits contamination of CVC cultures is unknown. Because the ISDD diverts the initial portion of the specimen (excluding blood with the heaviest burden of bacteria), the ISDD could result in exclusion of colonized catheters. Ideally, use of the ISDD would not decrease sensitivity while improving specificity (prevention of contamination due to bacteria on the connector). This in vitro study, modeling CVC intraluminal colonization and connector contamination, suggests possible clinical utility for ISDD CVC cultures.

In the colonization study, standard cultures and ISDD cultures detected 100% of colonized CVCs. Because the ISDD excludes the initial more heavily colonized portion, there was a longer TTP for ISDD cultures compared to standard cultures (2.49 hours longer incubation for first bottle, 1.22 hours longer for second bottle). This is supported by the greater number of bacteria present in quantitative cultures (1.17 × 10^5^ CFU/mL (ISDD) vs 3.96 × 10^6^ CFU/mL (standard)). The longer TTP noted with ISDD cultures would generally not be regarded as clinically significant. Thus, the ISDD method detects colonized CVCs without loss of sensitivity.

The utility of the ISDD technology for CVC cultures comes with improved specificity. The contamination study, which modeled low-level contamination of CVC connectors, demonstrated a decrease in blood culture contamination from 36% to 16% (56% relative risk reduction). This observation did not reach the level of statistical significance (*P* = 0.196) most likely due to study size/statistical power. The low inoculum level for the contamination model is supported by clinical studies examining connector contamination in which most contaminated connectors exhibited only a few CFU.^10^ At our center, despite robust infection prevention and stewardship programs, blood culture contamination results in an extra day of hospitalization and antibiotic treatment.^2^ Additional adverse effects include unnecessary laboratory studies, unneeded antibiotics, diagnostic confusion, and unnecessary removal of CVCs.^3,8,9^

The CVC colonization model resulted in a heavily colonized intraluminal surface from a small inoculum (10 CFU) after a short incubation time (24 hours) in the presence of a rich nutritional environment. This is analogous to a CVC used to instill total parenteral nutrition, lipids, or blood products. The contamination model mimics the small inoculum associated with contamination of the catheter hub or connector and less frequently resulted in positive cultures (36%) and rarely was detected by quantitative culture (8%). The dose-ranging study indicated that at higher levels of connector contamination, the discriminatory effect of the ISDD was lost. However, we believe low-level inoculum is a more accurate reflection of clinical conditions.^10^

In this CVC contamination/colonization model, the ISDD resulted in 100% sensitivity and improved specificity and justifies a prospective clinical trial.

## Supporting information

Rupp et al. supplementary material Rupp et al. supplementary material

## Data Availability

All raw data are available upon request.

## Supplementary material

The supplementary material for this article can be found at https://doi.org/10.1017/ice.2024.220.
