# Supplementary material for: In vitro assessment of effect of initial specimen diversion device on detection of central venous catheter contamination or colonization
Source: Infect Control Hosp Epidemiol. 2024 Dec 19;46(2):197–200. doi: 10.1017/ice.2024.220 (PMC11790329; doi:10.1017/ice.2024.220)
Supplement: Rupp et al. supplementary material [file S0899823X24002204sup001.docx]

**Methods**

Central venous catheters (CVC): Arrow triple-lumen, 7 French (2.5 mm OD) x 20 cm (Teleflex).

Catheter connector: MaxZero^TM^ needleless connector (Becton Dickinson).

Bacteria*: ica-* positive, biofilm-producing *Staphylococcus epidermidis* expressing green fluorescent protein (GFP) (1457/pCM29) as previously described [1].

Initial specimen diversion device (ISDD): SteriPath blood collection system (Magnolia Medical Technologies).

Catheter colonization: Catheter colonization was simulated by filling the distal lumen of the CVC with Difco Trypticase Soy Broth (TSB, Becton Dickinson) containing 10 colony forming units (CFU) of *S. epidermidis* 1457/pCM29. The CVCs, maintained in a sterile catheter contamination shield (Cath–Gard, Teleflex), were incubated overnight at 37°C. 50 CVCs were sampled by either standard blood culture or ISDD method.

Catheter contamination: The distal lumen of the CVC was pre-filled with sterile phosphate-buffered saline (PBS), and catheter connector contamination was simulated by inoculation of 10 CFU of *S. epidermidis* 1457/pCM29 in 50 µL of TSB onto the diaphragm of the connector valve attached to the distal lumen of the CVC. The inoculum was allowed to air dry for 60 minutes at room temperature. 50 CVCs were sampled by either standard blood culture or ISDD method.

Blood culture: Blood cultures were simulated by drawing sterile PBS through the distal lumen of the CVC. In the standard blood culture method, the ISDD was pre-engaged (diversion chamber closed) and then connected to the distal lumen of the CVC. For the ISDD method, the ISDD was connected to the distal lumen of the CVC and engaged to divert and sequester the initial 1.5-2 mL of PBS that was drawn through the CVC. For both methods, a Vacutainer tube (Becton Dickinson) was used to collect the next 1 mL of PBS for quantitative culture. Two 10 mL samples were then collected into two BACTEC Plus Aerobic/F blood bottles (Becton Dickinson).

The bacterial titer in CFU/mL was determined from the colony counts on TSA plates incubated overnight at 37°C. Blood bottles were incubated in a BACTEC FX instrument (Becton Dickinson) and monitored for up to 5 days per Nebraska Medicine Clinical Microbiology Laboratory protocol. Time-to-positivity (TTP) was recorded. All colonies obtained from quantitative cultures and from streaking of positive blood bottles were confirmed to express GFP.

Dose-ranging preliminary study: To establish inoculum levels to simulate CVC connector contamination and catheter colonization, a dose-ranging study was conducted using 3 CVCs per inoculum group: 10^1^ CFU-10^3^ CFU in colonization model;10^1^ CFU-10^4^ CFU in contamination model.

Scanning electron microscopy: Samples for SEM were fixed in 2% glutaraldehyde, 2% paraformaldehyde in 0.1M Sorenson’s phosphate buffer (pH 6.2) for ≥24 h at 4°C. Samples were washed three times with phosphate buffer and post-fixed in 1% aqueous solution of osmium tetroxide for 30 minutes. Samples were dehydrated in a graded ethanol series (50%, 70%, 90%, 95%, 100% x3), critical point dried, attached to aluminum SEM stubs, and silver paste was applied. Samples were coated with ~50nm Gold-Palladium alloy in a Hummer VI Sputter Coater (Anatech USA) and imaged at 15kV in a FEI Quanta 200 SEM operating in high vacuum mode.

Statistical analysis: Descriptive statistics (means, standard deviations) were used for data summary. Fisher’s exact test was used to compare the proportion of positive cultures detected between the ISDD and standard methods for contaminated and colonized CVCs. The Wilcoxon rank sum test was used to compare the median CFU per mL between the two blood culture methods for contaminated CVCs and for colonized CVCs. All analyses were done using SAS, Version 9.4.

Sample Size: We anticipated that essentially all blood cultures from the colonized catheters would be positive and thus sample size was not based on colonized catheters but on contaminated catheters. PASS software was used to calculate differences that could be detected between the methods with 80% power in the contaminated catheter model. We calculated that 10 contaminated catheters in each group would yield 80% power to detect a difference between group proportions of 0.45. The proportion of positive cultures in group 1 (diversion device) was assumed to be 0.99 under the null hypothesis and 0.54 under the alternative hypothesis. The proportion of positive cultures in group 2 (standard method) was 0.99. The significance level of the test is 0.05.

1. Roy P, Horswill AR, Fey PD. Glycan-Dependent Corneocyte Adherence of Staphylococcus epidermidis Mediated by the Lectin Subdomain of Aap. mBio. 2021 Aug 31;12(4):e0290820. doi: 10.1128/mBio.02908-20. Epub 2021 Jul 13.
